# A Qualitative Study on Parenting Practices to Sustain Adolescent Health Behaviors in American Indian Families

**DOI:** 10.3390/ijerph20217015

**Published:** 2023-11-03

**Authors:** Christine Hodgson, Dylan Decker, Teresia M. O’Connor, Melanie Hingle, Francine C. Gachupin

**Affiliations:** 1 Family Health Care Nursing, University of California San Francisco, San Francisco, CA 94143, USA; christine.hodgson@ucsf.edu; 2 Cancer Center Division, University of Arizona, Tucson, AZ 85721, USA; dylandecker@arizona.edu; 3 USDA/ARS Children’s Nutrition Research Center, Baylor College of Medicine, Houston, TX 77030, USA; teresiao@bcm.edu; 4 School of Nutritional Sciences and Wellness, College of Agriculture, Life and Environmental Sciences, University of Arizona, Tucson, AZ 85721, USA; 5 Department of Family and Community Medicine, College of Medicine, University of Arizona, Tucson, AZ 85721, USA

**Keywords:** health promotion, disease prevention, diabetes, obesity, wellness, parenting, American Indian

## Abstract

American Indian (AI) adolescents who practice healthy behaviors of sleep, nutrition, physical activity, and limited screen time can lower their lifetime risk of diet-sensitive disease. Little is known about how AI parenting practices influence the health behaviors of youth. The objective of this qualitative study was to explore how a group of AI parents of youths at risk of disease influenced their youth’s health behaviors after a family intervention. A secondary objective was to understand the role of AI parents in supporting and sustaining health behavior change in their youths following the intervention. Semi-structured in-depth interviews were conducted with AI parents (*n* = 11) and their young adolescents, 10–15 years old (*n* = 6). Parents reported facilitators to how they enacted healthy lifestyle behaviors, including family togetherness, routines, youth inclusion in cooking, and motivation due to a health condition in the family. Barriers to enacting healthy behaviors included a lack of time, a lack of access to health resources, negative role modeling, and the pervasiveness of screen media. Three major themes about the role of AI parenting emerged inductively from the interview data: “Parenting in nontraditional families”, “Living in the American grab-and-go culture”, and “Being there and teaching responsibility”. The importance of culture in raising youths was emphasized. These findings inform strategies to promote long-term adherence to behavior changes within the intervention. This study contributes to public health conversations regarding approaches for AI youths and families, who are not well represented in previous health behavior research.

## 1. Introduction

American Indian (AI) adolescents in the United States (U.S.) have a higher risk of obesity, type 2 diabetes, cardiovascular disease, and some cancers, than the general population [1]. These disparate outcomes are modifiable by health behaviors, including a healthy diet, physical activity, quality sleep, and limiting screen time. Youths can lower their lifetime risk of diet-sensitive disease through the promotion of healthy behaviors. Current US guidelines for weight reduction interventions and the prevention of obesity-related chronic diseases among children recommend family-based behavior change to promote health [2]. However, behavioral interventions for AI youths must account for the complex sociocultural history of AI people in the U.S. and honor traditional beliefs and practices [1]. Little is known about how to best engage AI parents through culturally appropriate means to help them support their children towards healthy dietary intake, physical activity, quality sleep, and reduced screen time.

Parenting practices are specific behaviors parents and guardians use to teach and socialize their children. Parenting practices have been targets of past health promotion interventions in the general population to promote a healthy weight and reduce the risk of obesity-related chronic diseases among children [3]. There is little published research on American Indian (AI) parenting practices to promote a healthy diet or physical activity, so the effects of parenting on AI youth health outcomes are unknown. Some culturally tailored studies about AI parenting have focused on youth mental health outcomes that may inform interventions for other health outcomes [4]. Goodkind et al. [5] implemented a weekly evening psycho-educational intervention with AI youths and their families over six months. The intervention was based on healing from historical trauma, reconnecting to culture, parenting/social skills building, and equine-assisted activities. Although parenting outcomes were not reported, the youths showed increased self-esteem, cultural identity, coping strategies, quality of life, and social adjustment. The project was shown to be feasible and acceptable with challenges on participant retention. Kulis et al. [6] performed a randomized controlled trial involving a 10-session parenting curriculum intervention with 575 parents of AI youths (ages 10–17), who were randomized to the intervention or a control group. Kulis et al. found significant effects on parenting skills and family functioning in the intervention group compared to the control group. Neither Goodkind et al. nor Kulis et al. addressed youth health behaviors on nutrition, sleep, physical activity, limiting screen time, or physical health outcomes in their parenting studies. Another study tested six existing parenting and family function scales in 606 AI parents across three tribes and found that the scales did not capture the lived experiences of AI parents [7], highlighting potential important differences in parenting within AI families that need to be considered in health promotion interventions.

The Achieving American Indian Youth Energy and Mental Health Balance (AYEM-B) intervention grew from a decades-long partnership between several southwestern U.S. tribes and a university multidisciplinary healthcare team [8]. AYEM-B was a residential camp intervention for AI young adolescents (ages 10–15) (referred to as youths) who were identified as being at risk of diet-related diseases. In 2019, the team received additional funding to enhance the intervention by adding booster sessions after the week-long residential camp and adding parenting components to the interventions to help support the youths in making behavior changes. Concurrently to this change, the 2019 COVID-19 pandemic occurred resulting in the intervention being modified to a Camp in a Box delivered to the whole family during a one-week intensive period of activities for the family to perform at home in the summer, followed by eight booster session packages delivered by mail every couple of weeks [8]. The scarcity of recommendations for AI parents regarding supportive parenting practices in the context of eating, physical activity, sleep, or screen use, in addition to measurement tools to measure these parenting practices, suggested the need for formative research to explore how and why parents influence their youth’s health behaviors. The primary objective of this qualitative study was, therefore, to explore *how* this group of AI parents influence and shape their youth’s nutrition, sleep, physical activity, and screen time health behaviors, with a focus on facilitators and barriers. A secondary objective was to understand the *role* of AI parents in supporting and sustaining health behavior change, following family participation in the AYEM-B Camp in a Box.

## 2. Materials and Methods

### 2.1. Study Design

This study used a qualitative descriptive design; a naturalistic inquiry that stays close to the data and the surface of the words and provides a straightforward description of phenomena [9]. Due to limited extant research about parenting among AI families in relation to health behaviors, inductive analysis allowed for themes to emerge from the voices of the participants.

### 2.2. Study Setting

The AYEM-B Camp in a Box was implemented over three consecutive years (2020, 2021, 2022), while COVID-19 restrictions were in effect and the previous residential youth summer camp was cancelled. The distanced format followed the residential camp activities, which included one week of intensive, self-guided education sessions, plus eight booster sessions. Briefly, the families were mailed the materials for the educational activities rooted in the context of AI culture. The families learned about making healthy dietary choices, increasing physical activity, getting the right amount of sleep, and limiting screen time, among other health topics. The parents were provided with information on how to engage in parenting practices that are believed to support their child toward adopting health behaviors relating to healthy eating, physical activity, sleep, and screen use [2,3,10]. All activities were designed to be experiential and engage all household members.

### 2.3. Ethical Considerations, Sampling, and Participant Recruitment

This study was approved by the University of Arizona IRB and the IRB of the Tribal partners and met the additional protections and safeguards for children and AI populations required by the IRB. Purposive sampling [11] identified parents/caregivers or youths that could articulate their experiences around parenting AI youths concerning health behaviors. The parent inclusion criteria were being a parent of AI ancestry (mother, father, or primary caregiver who serves in a parent role for a youth of AI ancestry) who had taken part in the AYEM-B Camp in a Box and already met the larger study requirements [8]. The adolescent inclusion criteria were being an AI youth aged from 10 to 15 years old who had participated in the AYEM-B Camp in a Box. For the remainder of this article, the word “parent” represents every person who was interviewed with a parenting role for their youth, whether they are a mother, father, grandparent, or other primary caregiver.

### 2.4. Data Collection

The multidisciplinary research team developed a semi-structured interview script over several meetings. The questions pertained to the participant’s thoughts about parenting a youth, the successes and challenges of enacting behaviors learned as part of the intervention, and feedback about the AYEM-B Camp in a Box program. An example of a parent interview question was, “What challenges have you experienced in trying to encourage your child to eat healthy?” An example of a youth interview question was, “How, if at all, does your parent or parents influence or guide you regarding how and what you eat?”

The first interviewer (CH) administered the interviews over the phone with five parents in May 2021. Following the first cohort of interviews, the interview script was modified for youth participants. A second interviewer (DD) conducted interviews in June 2022 and October 2022. All participants attended the AYEM-B Camp in a Box program in the previous year.

### 2.5. Data Analysis

Conventional content analysis was used because of the flexibility of the method and the lack of existing theory and research about AI parenting support for youth health behaviors [12]. The coding was an iterative process that began during the first interview using open-ended questions and probes or prompts as needed, with the interviewer CH taking notes and writing down the first impressions. When the transcripts were analyzed, in vivo coding was implemented, examining each line for keywords or phrases. The codes were organized into manageable sections that were sorted and sifted to identify clusters and broader themes. A codebook was developed after the first cohort was interviewed and shared with the second interviewer, DD. During interviews with the second and third cohorts, DD used the same codebook and techniques to code all the transcripts, adding new codes when they emerged. All the transcripts and the cumulative list of codes were uploaded to the Dedoose software (v9.0.17, Los Angeles, CA, USA) and coded again by CH. The coding system allowed both interviewers to code all the transcripts. The research team discussed the data analysis and reached a consensus on the representation of the data. Coding was applied within each question and across all questions to gain the most insight about parenting. The codes specific to each interview question were organized into “facilitators” and “barriers” of each behavior: nutrition, physical activity, sleep, and limiting screen time. One hundred and thirty-two specific codes from across all the interview questions were grouped into 14 categories, out of which three overarching themes emerged.

Rigor was addressed using the criteria for trustworthiness established by Lincoln and Guba [13]. Credibility was assured by sampling three different cohorts of participants and through a careful interview script development and interview process. Dependability was addressed using a detailed track record of the data collection process and a stepwise application of the interview process with each cohort. Confirmability of the findings required that authors used reflexivity to acknowledge their bias and experiences. The following section reports on researcher reflexivity.

### 2.6. Researcher Background and Positioning

The research team acknowledges how researchers’ professional and personal life experiences affect the objectivity of the findings from qualitative research [13]. The first interviewer (CH) identifies as a white, middle-aged nurse practitioner with experience working in a school-based health clinic on a Northern Plains reservation. She is a wife and mother of three older adolescents. The second interviewer (DD) is an AI young adult and post-baccalaureate student, who has spent most of their life in a reservation. DD has work experience in behavioral health and emergency medical health services in an AI reservation. The reflexivity of these authors was a key factor in the research analysis, including frequent discussions on how each author perceived the data through different philosophical lenses.

## 3. Results

The participants in this study consisted of eleven parents, six of whom had their youths participate in an interview. The eleven adult parents or caregivers (all female) and six youths, between the ages of 10 and 15 years old (83% female), participated in the AYEM-B Camp in a Box intervention in either 2020 (*n* = five parents), 2021 (*n* = two parents; *n* = three youths), or 2022 (*n* = four parents; *n* = three youths). Eight of the eleven parents/caregivers were mothers and three were grandmothers raising their grandchildren. Some youths were hard to engage in conversation despite the use of probes and prompts. Five youth interviews lasted between 18 and 24 min, while one youth interview was only 12 min long. The average length of youth interviews was 19 min, compared to the average length of parent interviews of 31 min. Correspondingly, text extractions from the eleven parent interview transcripts were greater in number than from the six youth interviews (667 coded excerpts derived from the 11 parent interviews, and 125 coded excerpts derived from youth interviews, respectively). The findings from the youths corroborated the findings from the parents. Because of the abundance of parent-associated excerpts and codes relative to youth-associated excerpts and codes, the youth interviews did not provide any new or different information. However, the findings from the youths did corroborate the findings from the parents.

The findings associated with the first objective, how AI parents influence and shape their youth’s health behaviors on nutrition, physical activity, sleep, and limiting screen time, are summarized in Table 1. The findings are reported as facilitators and barriers to enacting each health behavior: the results combine the parent and youth data, while distinguishing between parent or youth responses when it is important for context. Common facilitators for health behaviors included family togetherness, routines, including youth involvement in cooking or planning activities, motivation due to a health condition in the family, and teaching children about health behaviors. Common barriers to health behaviors included a lack of time, a lack of access to healthy foods or environments for activities, negative role modeling, and the pervasiveness of screen media.

**Figure 1:**
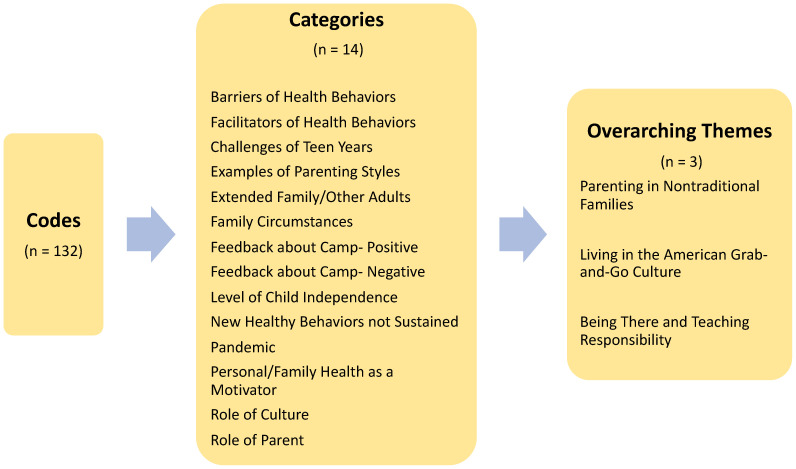
Codes, categories, and themes associated with the role of American Indian parents in supporting healthy behaviors in youths.

The coding across all the interview questions revealed patterns related to the second objective, to understand the role of AI parents in supporting and sustaining healthy behavior change following a family’s participation in the AYEM-B Camp in a Box. Three themes emerged inductively from the codes and categories that help explain the “how” and the “why” related to the role of AI parents in supporting their youth’s healthy behaviors (see Figure 1).

### 3.1. Theme 1: Parenting in Nontraditional Families

Unprompted, seven mothers, one grandmother, and one youth shared stories on experiencing a single parent household. In contrast with a “traditional family” in the Euro-Western sense, which refers to a nuclear family model with a mother, a father, and child(ren), the study participants were all female and mostly single parents. The women took on all the parental roles in the absence of a father figure in the household. A grandmother stated, “Well the father really hasn’t been around for these kids since they were little. You know he has been going in and out of jail, so he hasn’t really been [around] and like we’ve been trying to do both for them. Being a mother and a father”. It should be noted that when prompted to explain their thoughts on whether fathers and mothers had similar parenting roles, the adult and youth participants generally stated that the responsibilities of both parents were equally important. They described ways in which mothers and fathers individually could make different contributions to the household.

Extended family played a major role for the participants. Many relied on grandparents, such as a parent who said, “It is me, and then there [are] her grandparents, which is my parents, [they] play a big role in raising her”. Other important people mentioned by the youths in the interviews were: “A nina or nino”, a parent’s “best friend”, “a godmother”, and “aunts and uncles”. One youth told of the importance of their siblings, “…because if you have older siblings, they can help you through if they’ve been through it already”. One parent commented on extended families, “They’re like the backups. If I can’t do it then, you know, I have to rely on somebody to either… take her to school, to any type of program or anything going on at school”.

A grandmother participant noted challenges in raising her grandchildren. She reported support from extended family as, “Yeah. They help, especially my brothers and my sisters. You know, they always talk to the kids, and they tell them, you know, ‘You gotta help your Grandma. She’s not that young anymore. Help her do things. Be there for her’”. One mother participant commented on the growing number of families she sees who are parented by grandparents and shared her concerns that, “I think when (the youth) were in a situation that’s not good and that’s the reason why they’re being put with grandparents and grandparents are trying to do the best that they can. And they’re trying to set rules when they didn’t have rules with them before. So that’s a little bit harder for the grandparents”. The same mother suggested, “And I think if they had some type of guidance to help them along the way, too. Cause sometimes like since they didn’t have the group of kids in the home. They’re not used to making an abundance of food, or having chores done, you know separately around the house, or setting rules. Because they were by themselves”. A different parent applauded grandparents for teaching their youths, “about like cultural stuff or traditions and things like that”.

Community and culture were topics that were not specifically included in the interview script but emerged in conversations as sources of strength. Three parents mentioned the importance of community resources, such as an Indian center or an Indian youth program for their youths to take part in activities after school. One mother shared how her family was learning their Indigenous language and about traditional stories and stated, “Knowing where you come from, your background. And that helps you as a person. I believe that it helps you and it makes you stronger and it makes you more proud. And you carry yourself …a lot higher when you know where you’re coming from”. She added, about traditional ways of living, “It would be nice to see that a lot more of that like in the tribal leaders”.

There was evidence of other variations in family experiences among the participants. One parent mentioned living on a reservation and another shared how they visited extended family on a reservation. Both women described a connection to nature and open space, where their youths could run and ride a bike and chase their dogs. Two families described households of seven or eight members; one parent described her child with special needs who had sensory issues and a developmental delay. One parent mentioned a recent loss in the family and how their grieving affected their health behaviors for some time. Despite a diversity of challenges, the participants were perceived as positive and strong by the interviewers. Congruently, the codes and categories supported a sense of thriving in this sample of youths and parents.

### 3.2. Theme 2: Living in the American Grab-and-Go Culture

The participating families reported having very busy lives, which influenced all the health behaviors supported by the AYEM-B Camp in a Box intervention: nutrition, physical activity, sleep, and limiting screen media. The theme of being busy was the most common barrier to healthy nutrition and physical activity in this study. One parent stated, “We do a lot of fast food, so we aren’t very healthy at all”. Another parent shared, “So, the challenges are mostly me, I guess, …we like eating salads, but it’s having to put—you know, making the time to go and purchase all the things that we need or, you know, just to eat healthy”. Regarding sleep, one parent said, “Like how I said we have to eat dinner late, so …they’ll be tired, but I’ll tell them, like, no, you can’t go to sleep, you just ate, so let your stomach settle. And so I think that’s one of our big issues right now, is we are forced to eat dinner so late just due to our schedule”.

Parents reported working late, working an evening shift, having long commutes to work or to school, and being tired. Some youths also had jobs that kept them from getting enough sleep or having family meals. Coordinating schedules for different family members was a notable challenge. Running out the door without having breakfast, grabbing fast food for lunch, and getting home too late to cook dinner were mentioned. Later dinners pushed bedtime back for a few families, affecting their sleep. Busy schedules left little time for physical activity. One parent said, “I’m always busy, busy, busy. And then, I have to go pay bills, or have to go get groceries, or I have to go—I’m always having to do something”. A couple of parents described their youths as being on their screens or phone “all the time” or “constantly”. Another parent described how it was a routine for her youth to be on the phone while in the car. Screen time was a common barrier to good quality sleep, but most parents reported some type of structure or rules around screens in the home. The interconnectedness of health behaviors was apparent.

### 3.3. Theme 3: Being There and Teaching Responsibility

When youths were asked, “Using your own words, what does it mean to be a parent?” The youths answered with the following short statements: “Being there for me”, “Be supportive”, “Take care of their kids, provide for them and just kind of push them to success”, “Being a parent is taking care of your kids and doing what is best for them”, “Take care of your child and provide for your child and be there for your child”, and “To take care of their kids and provide for them and just always kind of push them to success”. Follow-up questions allowed for some elaboration on the theme of “Being there” and the youths added, cooking for them, taking them to school and activities, talking to them, supporting them through school, and encouraging them to go to college. The parents’ answers echoed and elaborated on the youths’ responses about parenting roles.

Teaching their youths to be respectful and responsible was prioritized according to both youths and parents. Youths described responsibility as teaching “right from wrong” or “good from bad”, and teaching life skills. Parents used the words “responsibility”, “mutual respect”, “respectful”, and “helpful” to others. Chores and work around the house were important to many participants. One grandparent stated, “I’m not saying they are the best kids, but they really are good kids. You know? They help. Um like if I’m doing something and like my grandson or my granddaughter says grandma can I help you? You know, and they are really good about things, and they are very respectful”. A common thread for the majority of parents was having clear expectations and boundaries for their youths related to healthy behaviors. Most youths and parents mentioned the importance of success in school. Culture was mentioned in the context of raising youths, by one parent who said, “Setting a good example, and …just the traditional teaching, and a lot of that now, is not, it’s not really practiced, and it’s not pushed”.

### 3.4. Feedback about the AYEM-B Camp in a Box

The last two questions in the interview script guided the interviewers to ask for feedback about the AYEM-B Camp in a Box intervention. The responses from all 17 participants were positive, highlighting the novelty and fun involved in the intervention. Positive aspects about the AYEM-B Camp in a Box included prompting families to be accountable to one another and program staff, providing a structure and schedule for activities, and bringing families closer. Youths and other children in the family (siblings, cousins) looked forward to receiving the boxes in the mail and really liked the healthy snacks and arts and crafts. Only a few participants had suggestions for improvements, while many said they would like more of a particular activity or exercise. One parent summarized, “I think you guys did a pretty good job. I think we hung on to most of the stuff. We learned a lot last year. I think, really, it’s just up to us to implement it throughout the year. And we’re off and on, but we’re trying to learn a lifestyle so, it’s just going to take longer, you know?”

A youth shared, “I also think that more of the hands-on activities, like painting. Like, last year what we did was paint, like, the painted rocks and stuff. We did all that and, like, all of our family did that and it kind of just brought us, like, a lot closer, because at first we were all annoyed with each other and were all, like, getting mad at each other, arguing, but we all just kind of settled and all because of the Camp in a Box. And that’s really—that’s what really brought us together. And there was more understanding of each other”.

## 4. Discussion

### 4.1. Main Findings

This study extends the understanding on parenting among AI families related to helping youths maintain behaviors learned through a lifestyle behavioral intervention. Common facilitators and barriers to healthy nutrition, physical activity, sleep, and screen media use, were identified for AI families, which were similar to a 2008 study of Navajo parents of preschool children [14]. The challenges of limited time, the availability of healthy foods, and parenting preferences were reported by Cunningham-Sabo et al. and reverberated here, as were the facilitators to social and school support. The current study allowed for parents and youths to voice their opinions and explain how the AYEM-B Camp in a Box booster sessions might be structured to help sustain healthy family behaviors in the long term. Booster sessions can provide reinforcement on quick healthy meals and preparing meals ahead of time. Healthy habits were easy at first, but became hard to maintain over time for the participants, suggesting a possible role for a small reminder to families after the booster sessions are complete.

Qualitative themes on parenting practices were identified. Like the existing literature, culture was reported as an important factor in AI parenting [4,5,6], as well as the extended family and parental role modeling [4]. The theme about nontraditional parenting suggests the participants have unique challenges and strengths, which should be considered in future programs. For example, in a recent study of 61 parent/child dyads equally distributed across six racial/ethnic minority groups (including AI), researchers found that parenting stressors, a depressed mood, and interpersonal conflicts were associated with less healthy food parenting behaviors in AI families, including fewer homemade meals [15]. Supporting families through the psychosocial challenges of single parenting and grandparents raising children may ultimately improve the healthy behaviors of their youths.

### 4.2. Strengths and Limitations

A strength of this study was that it was grounded in AI culture. The literature suggests that culturally tailoring instruments and interventions in tribal research programs will improve the validity of the studies [1,4,5,6,16]. The interview guide was developed by AI researchers and their multidisciplinary team after decades of experience working with AI youths in the context of youth wellness and health promotion. Another strength of this study was the spotlight on the voices of youths and parents. Oftentimes, programs are administered without input or consideration of the needs of the population of study.

The relatively small convenience sample of parents (*n* = 11) and youths (*n* = 6) completing the interviews limits the breadth and generalizability of the findings. Themes just began to emerge but did not reach saturation, the point where new themes stop emerging [17]. The parents interviewed were from one of three southwestern U.S. tribes and the findings may not be relevant to other AI populations. Another source of acknowledged bias is that the authors and interviewers gathered and understood the information through the lens of their own experiences, knowledge, and worldviews. The authors did engage in self-reflective assessments at all stages of the study to reduce the potential of imposing their view on the data. Discussions with the team during coding and analysis were also helpful for reflexivity [13].

### 4.3. Relevance of the Study and Policy Implications

The findings from this study will inform the continuing AYEM-B interventions, as the program shifts back to a residential one-week program for youths. Booster sessions will continue to engage the entire family in healthy behaviors. This study contributes to conversations regarding public health strategies and approaches for AI youths and families, who are not well represented in previous health behavior research. Our study suggests the need to consider variations in family structure, the social determinants of health, and the role of culture in future research or policy formulation.

## 5. Conclusions

Data from interviews with parent and youth AYEM-B Camp in a Box participants offered new knowledge about parenting among AI families, facilitators, and barriers to helping their youths enact healthy behavioral change, as well as ideas about how to improve future health promotion interventions and booster sessions for AI families. The intervention was positively perceived by parent and youth interviewees, including interviewees expressing gratitude for bringing their family members closer together. This qualitative study also offers guidance for further refinement of the AYEM-B intervention to promote long-term adherence to behavioral changes. The findings provide an example of how AI parents are raising their young and supporting healthy behavioral change around nutrition, physical activity, sleep, and limiting screen media use.

## Figures and Tables

**Table 1:** Parent influence on youth health behaviors taught by AYEM-B Camp in a Box.

	Facilitators	Barriers
Nutrition	Including youth in meal planning, shopping, preparation, home-cooked meals	Lack of time to cook, eating on the run, and eating out Parent: “We’re pretty much on the go a lot. So, it’s kinda hard at times for us sometimes to get… healthy, good, cooked meal. Sometimes we just got to grab things on the way home.”
	Trying new foods and increasing vegetables Parent: “He really likes to cook. He likes learning new recipes and cooking them. So, in that case, I will say, Okay, let’s try to find something you can cook that includes vegetables or something new.”	Parent role model Parent: “What I eat, she eats. So, I guess I’m the lead on that.” Youth from a different family: “I just think, usually what my parents eat, I eat. Kind of like the same.”
	Family meals	Hard to change old habits, cravings for “junk food” or soda
Physical Activity	Motivated by health conditions of parent or family member Parent: “You don’t want to be told that you are diabetic… so to make the healthy choices now.”	Environmental conditions, such as apartment living, summer heat, lack of access to parks, pandemic restrictions
	Being active together as a family, such as walking, sports, dancing, chores	Asthma in youth or parent
	Home equipment, such as a treadmill	
	School gym class and extracurricular sports	Parent role model Parent: “I think it starts with home. You gotta be able to be active yourself so their kids can see that… you’re trying to get them involved with what they’re doing because if you’re not doing it, they’re not gonna do it.”
Screen Media	Parent monitoring time on screens Youth: “It’s like my mom doesn’t want us to just sit around and play on our phones and on the games…she’ll tell us, like ‘Hey. You need to clean your rooms’ and telling us we should do some-thing for, like, an hour of doing something outside, or even coming inside and cleaning up with doing chores, other than just being on our phones.”	Challenging teen attitudes Parent: “The attitude. The attitude of, well, everyone else is doing it. Why can’t I, type of attitude. Then, no, oh, I want to look at these videos, or I am bored.”
		Pervasiveness of screens Youth: “I would say that it’s kind of, like, a problem with us, like, try not to have so much screen time so much.”
	Watching shows or movies as a family	
	Parent monitoring content of screen time and social media	
Sleep	Routines and bedtimes Youth: “My mom will walk around saying “You guys need to wake up” and usually that’s how it goes.”	Parents’ work schedule
		Parents go to bed before youth
	Teaching youth about sleep Parent: “If you don’t get enough sleep then you’re not gonna do good on your test, cause you’re gonna be too sleepy trying to think about what your questions are and stuff like that.”	Screens at bedtime Parent: “Yes, yes, that’s what I noticed, for a while where I didn’t, and then I got complaints from teachers, so and so is falling asleep, so and so, and it was just like, oh no, no, then I had to start taking that away from them. And I tell them it’s a privilege, that’s a privilege and I can take that away whenever.”
		Teens stay up late, sleep in late on weekends

## Data Availability

The AYEM-B data are owned by the respective participating sovereign tribal nations. The data are not publicly available, and all data requests must be reviewed and approved by the tribes.
